# Photodynamic Therapy with the Silicon Phthalocyanine Pc 4 Induces Apoptosis in Mycosis Fungoides and Sezary Syndrome

**DOI:** 10.1155/2010/896161

**Published:** 2010-12-12

**Authors:** Minh Lam, YooJin Lee, Min Deng, Andrew H. Hsia, Kelly A. Morrissey, Chunlin Yan, Kashif Azzizudin, Nancy L. Oleinick, Thomas S. McCormick, Kevin D. Cooper, Elma D. Baron

**Affiliations:** ^1^Department of Dermatology, University Hospitals of Cleveland, Case Western Reserve University, 11100 Euclid Avenue, Lakeside 3500, Cleveland, OH 44106-5028, USA; ^2^Department of Dermatology, Huashan Hospital, Fudan University, Shanghai, China; ^3^Department of Radiation Oncology, Case Western Reserve University, 10900 Euclid Ave., Cleveland, OH 44106-4942, USA; ^4^Dermatology Department, Cleveland Veterans Affairs Medical Center, Cleveland, OH 44106, USA

## Abstract

Our current focus on the effects of Photodynamic Therapy (PDT) using silicon phthalocyanine Pc 4 photosensitizer on malignant T lymphocytes arose due to preclinical observations that Jurkat cells, common surrogate for human T cell lymphoma, were more sensitive to Pc 4-PDT-induced killing than epidermoid carcinoma A431 cells. Mycosis fungoides (MF) as well as Sezary syndrome (SS) are variants of cutaneous T-cell lymphoma (CTCL) in which malignant T-cells invade the epidermis. In this study, we investigated the cytotoxicity of Pc 4-PDT in peripheral blood cells obtained from patients with SS and in skin biopsies of patients with MF. Our data suggest that Pc 4-PDT preferentially induces apoptosis of CD4^+^CD7^−^ malignant T-lymphocytes in the blood relative to CD11b^+^ monocytes and nonmalignant T-cells. *In vivo* Pc 4-PDT of MF skin also photodamages the antiapoptotic protein Bcl-2.

## 1. Introduction

Photodynamic therapy (PDT) involves exposure of tumors to a photosensitizing drug followed by irradiation with light of an appropriate wavelength. PDT is effective in the treatment of cancers of different organ systems including the lungs, bladder, gastrointestinal tract, and skin. Cutaneous neoplasms serve as ideal targets of PDT due to their accessibility. Moreover, PDT of skin lesions provides the opportunity to utilize optical instrumentation that could monitor treatment parameters in real time [1] and potentially capture mechanistic events occurring in the tumor *in vivo*. In dermatology, PDT is approved for the treatment of actinic keratoses but has been used off-label to treat basal cell carcinoma, Bowen's disease, and mycosis fungoides [2]. The interaction of photosensitizer, light, and oxygen results in oxidative stress that destroys the pathologic cells. Due to the prolonged skin photosensitivity that systemic photosensitizers such as Photofrin could cause, topical agents such as 5-aminolevulinic acid (ALA) and its derivatives are preferred [3] in the PDT of cutaneous disease. ALA-PDT, however, has unwanted side effects such as pain, irritation, and edema during and after treatment. Due to its limited penetration into the skin, ALA is effective in treating lesions only 2-3 mm deep [4]. ALA also requires an incubation period between application and light exposure to allow its metabolic conversion into the active photosensitizer protoporphyrin IX (PpIX). Thus, development of other topical PDT drugs with better safety and efficacy profiles is warranted. 

The silicon phthalocyanine Pc 4 [HOSiPcOSi(CH_3_)_2_ (CH_2_)_3_N(CH_3_)_2_] is a second generation photosensitizer that more efficiently absorbs red light at a longer wavelength (*λ* = 675 nm; *ε* = 2 × 10^5^) than PpIX (peak furthest to the red *λ* = 630 nm; *ε* ≈ 5 × 10^3^) [5]. Pc 4 binds to mitochondria, endoplasmic reticulum (ER), Golgi, and perinuclear membranes [6]. Photo-irradiating cells with 675 nm light after treatment with Pc 4 generates reactive oxygen species (ROS), for example, singlet oxygen, which leads to instantaneous modifications of biological molecules, such as lipids and proteins [7–9]. Within minutes to hours, this can lead to necrosis as well as the activation of programmed cell death or apoptosis [10]. Secondary local and systemic events such as inflammation and/or antitumor immune responses occur within days or months, depending upon cell type or tumor treated, as well as on the photosensitizer and light regimen used [11].

PDT-dependent cellular protein modifications may include protein oxidation, cross-linking, and degradation [9, 12–14]. Because these phenomena occur in an early stage of PDT, they may serve as a useful marker for early apoptotic events. As an important regulator of apoptosis, the mitochondrial membrane protein Bcl-2 functions to block apoptosis by inhibiting the release of cytochrome *c*. Pc 4-PDT, as well as PDT with a variety of photosensitizers, photodamages Bcl-2 with a dose-response that correlates with cell killing [12, 15]. Bcl-2 photodamage is characterized on western blots as the immediate nonenzymatic loss of the native protein and the formation of slower migrating complexes of Bcl-2 with neighboring proteins and lipids [16]. Another early marker of apoptosis is the Apo 2.7 antigen, a 38 kDa protein that is detected on the membrane of mitochondria when cells are in the early stage of apoptosis, suggesting that this protein may be involved in an early event of the molecular cascade [17]. Furthermore, the Apo 2.7 antigen is expressed in apoptotic cells but not in normal cells or cells that undergo death by nonapoptotic mechanisms, such as necrosis.

Our previous findings indicated that Jurkat cells were more sensitive to Pc 4-PDT-induced killing than epidermoid carcinoma A431 cells, while uptake of Pc 4 by both cell populations was shown to be similar in low to moderate doses (0.1 nM to 100 nM) [18], suggesting that Pc 4-PDT might be more effective in treating lymphomas than epithelial cancers. In both epidermoid and Jurkat cells, PDT with Pc 4 promotes the mitochondrion-mediated pathway of apoptosis, leading to eventual cell death [7]. The greater sensitivity of Bcl-2 to photodamage in Jurkat cells correlates with the greater response seen in those cells, indicating a role for Bcl-2 damage in the response in both cell types. These results suggest that Pc 4-PDT may offer a therapeutic advantage in targeting malignant T-cells of cutaneous T-cell lymphoma (CTCL) [18].

Mycosis fungoides (MF) is the most common type of CTCL accounting for almost 70% of all primary cutaneous lymphomas [19]. Sezary syndrome (SS) is the leukemic form of CTCL, involving the skin, lymph nodes, and peripheral blood. Patients with SS can have detectable levels of the leukemic T cells (Sezary cells) in their circulation, and the immunophenotype of these cells is often characterized by the expression of CD4 and absence of CD7 [20, 21]. In this study, we investigated the susceptibility of malignant T-lymphocytes that characterizes SS to the phototoxic effects of Pc 4-PDT *ex vivo, *and the effect of Pc 4-PDT *in vivo* to damage the antiapoptotic protein Bcl-2 in the skin lesions of MF.

Peripheral blood mononuclear cells (PBMCs) were obtained from patients with SS and treated with Pc 4-PDT. Apoptosis was detected (by positive staining with APO 2.7+) in the PBMC preparation, and levels of apoptosis observed in malignant CD4^+^CD7^+^ T cells *versus* normal bystander T cells and non-T cells were compared by flow cytometry. Pc 4-PDT treated skin biopsy specimens obtained from patients with MF were evaluated for changes and/or the level of Bcl-2, as an indication of apoptosis-related photodamage.

## 2. Materials and Methods

All procedures involving human subjects were approved by the Institutional Review Boards of Case Western Reserve University, University Hospitals Case Medical Center and the Veterans Affairs Medical Center.

### 2.1. PBMC Isolation and Preparation

PBMCs were isolated by Ficoll-Histopaque centrifugation from 60 ml of whole blood as described previously [22]. Samples were taken from three patients (age 67 to 82, 2M: 1F) with a diagnosis of SS confirmed by the presence of more than 10% CD4^+^CD7^−^ circulating cells and identification of TCR gene rearrangement. Cell viability was determined by trypan blue exclusion and was quantified using a hemocytometer.

### 2.2. Pc 4-PDT of PBMCs

PBMCs from each patient were cultured at 10^6^ per ml of complete medium (RPMI 1640 supplemented with 10% fetal bovine serum, 1% Pen Strep solution, and 1% L-glutamine) in 60 mm cell culture plates. Pc 4 (provided by Dr. Malcolm E. Kenney, Chemistry Department, Case Western Reserve University) was dissolved in N,N-dimethylformamide (DMF) to prepare a 0.5 mM stock solution. It was further diluted in complete medium to 20 nM, 70 nM, and 100 nM. The Pc 4-containing complete medium was then added to the PBMCs, which were incubated at 37°C for one hour. The PBMC cultures were irradiated using a LED array (*λ*_max_ ≈ 670–675 nm) at a fluence of 200 mJ/cm^2^ as previously described [8]. After irradiation, the cells were incubated from 16 to 18 hours at 37°C to allow sufficient time for apoptosis induction.

### 2.3. Flow Cytometry Analysis

Following Pc 4-PDT and overnight incubation as described above, PBMCs were characterized and phenotyped by flow cytometry. The total PBMCs were gated into three distinct populations (CD4^+^CD7^+^ T-lymphocytes, CD4^+^CD7^−^ malignant lymphocytes, and CD11b^+^ monocytes). FACS analysis was performed on an LSR II four-laser flow cytometer (Becton Dickson, Franklin Lakes, NJ). Antibodies used to identify the cell surface antigens were mouse antihuman CD4 (identified with PerCp), mouse antihuman CD7 (identified with FITC) (BD Pharmingen, San Jose, CA), and mouse antihuman CD11b^+^ (identified with APC) (Becton Dickson, Franklin Lakes, NJ). Levels of apoptosis induced by Pc 4-PDT in each population were characterized by permeabilizing the cells with digitonin and staining them with APO 2.7 PE-conjugated antibody (Immunotech/Beckman Coulter, Marseille, France).

### 2.4. Skin Biopsy Acquisition

Biopsies that were previously acquired from a Phase I clinical trial, consisting of three- to six-mm punch specimens of early stage MF, were utilized for this analysis. In the Phase 1 trial, Pc 4-PDT was performed on selected MF lesions, whereas other MF lesions in the same patient served as untreated controls. Biopsies of both the untreated and treated lesions were obtained 24 hours after Pc 4-PDT and stored for analysis. Complete results of this clinical trial will be published elsewhere (manuscript under review). For this particular experiment, frozen tissue samples were chosen from three patients whose lesions partially responded to Pc 4-PDT, and three who were clinical nonresponders.

### 2.5. Western Blot

Each tissue sample was homogenized using a Polytron blender, and 30–60 *μ*g of protein extracted from each sample was subjected to SDS-PAGE on Invitrogen NuPAGE Bis-Tris 4–12% Gels. The proteins were transferred onto nitrocellulose membranes, stained with Ponceau S to confirm the transfer, and incubated in blocking buffer (5% milk-TBST) for 48 hours at 4°C. Immunoblot analysis was conducted with purified hamster antihuman Bcl-2 monoclonal antibody (BD Pharmingen, San Jose, CA; 1:1000 dilution) followed by horseradish peroxidase-conjugated mouse antihamster secondary antibody (BD Pharmingen, San Jose, CA; 1:5000 dilution), and then developed using the Super Signal West Pico Kit (Super Signal West Pico, Pierce, Rockford, IL, USA). As a loading control, actin was detected using antiactin (Novus Biologicals, LLC, Littleton, CO).

## 3. Results

**Figure 1:**
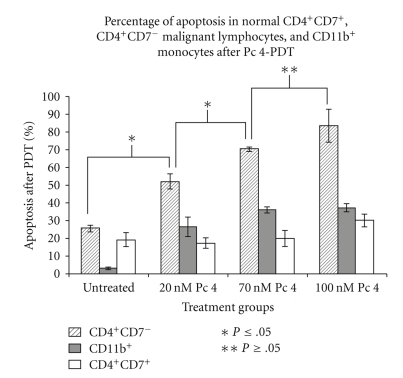
Pc 4-PDT dose response for the induction of apoptosis in CD4^+^CD7^−^ malignant lymphocytes, CD4^+^CD7^+^ normal lymphocytes, and CD11b^+^ monocytes of patients with Sezary syndrome. Peripheral blood mononuclear cells (PBMCs) were isolated by Ficoll-histopaque centrifugation and then treated with Pc 4 at 0–100 nM followed by photoirradiation (200 mJ/cm^2^).

**Figure 2:**
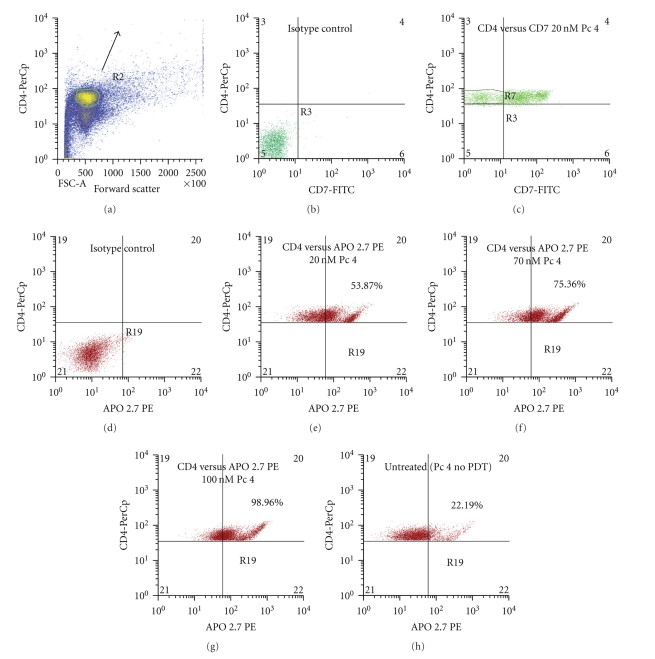
Representative flow cytometric histograms of the induced apoptosis of CD4^+^CD7^−^ lymphocytes treated with Pc 4-PDT. Total blood was obtained from patients diagnosed with Sezary syndrome. The histograms shown were taken from a single patient with SS. PBMCs were isolated, treated with Pc 4, and stained as mentioned previously in the Methods section. Lymphocytes were identified by their high CD4 expression (*y*-axis) and forward scatter (*x*-axis) as shown in yellow (region R2) in (a). CD4^+^CD7^−^ lymphocytes are shown in region R7 in (c). Levels of apoptosis in this population were analyzed in all three Pc 4-PDT doses. The data shown in (e), (f), and (g) exhibit a dose-dependent response in apoptosis induction among the three Pc 4-PDT concentrations. Levels of apoptosis in all three doses are significantly higher than in the untreated group shown in (h). Untreated cells were incubated with Pc 4 overnight but were not photo irradiated.

**Figure 3:**
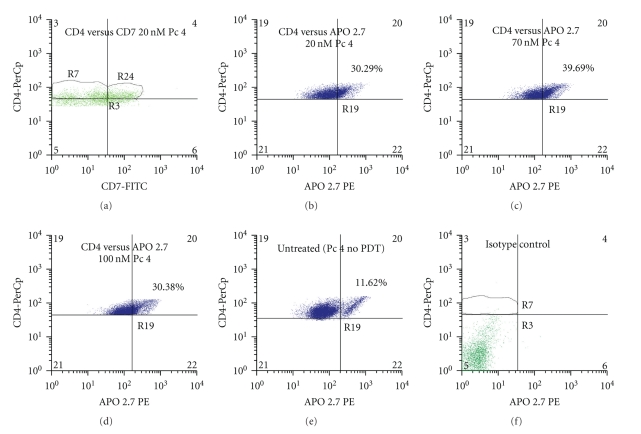
Representative flow cytometric histograms of the induced apoptosis of CD4^+^CD7^+^ lymphocytes treated with Pc 4-PDT. Total blood was obtained from patients diagnosed with Sezary syndrome. The histograms shown were taken from a single patient with SS. PBMCs were isolated, treated with Pc 4, and stained as mentioned previously in the Methods section. Lymphocytes were identified by their high CD4 expression (*y*-axis) and forward scatter (*x*-axis) as described previously. CD4^+^CD7^+^ lymphocytes were further identified in region R24 in (a). Levels of apoptosis induced by all three doses in this population were then analyzed. As shown in (b), (c), and (d), these levels remained relatively similar and *P*-values were largely insignificant from group to group. Apoptosis induction in the untreated group is shown in (e).

**Figure 4:**
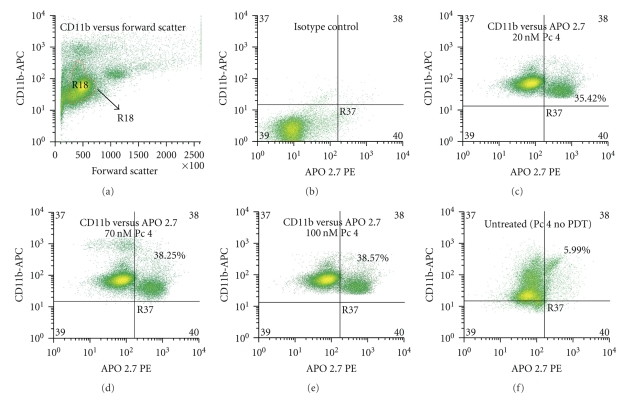
Representative flow cytometric histograms of the induced apoptosis of CD11b^+^ monocytes treated with Pc 4-PDT. Total blood was obtained from patients diagnosed with Sezary syndrome. The histograms shown were taken from a single patient with SS. PBMCs were isolated, treated with Pc 4, and stained as mentioned previously in the Methods section. Monocytes were identified by their high CD11b^+^ expression (*y*-axis) and forward scatter (*x*-axis) as shown in yellow (Region R18) in (a). Levels of apoptosis in all three Pc 4-PDT doses were then further analyzed from this population. As shown in (c), (d), and (e), levels of apoptosis were similar in the three treatment groups. Apoptosis induction in the untreated group is shown in (f).

 PBMCs from three patients with Sezary syndrome were exposed to Pc 4-PDT *in vitro*, and three subpopulations were evaluated for apoptosis 24 h later by staining with APO 2.7 and flow cytometry (Figure 1). The most striking observation was a marked PDT dose-dependent increase in apoptosis in the malignant CD4^+^CD7^−^ T lymphocytes. The average level of apoptosis in the malignant cells increased significantly from 25% of the untreated cells to 51.9%, 70.4%, and 83.6%, following irradiation of cells given 20, 70, and 100 nM Pc 4, respectively. This result is in sharp contrast to the levels of apoptosis exhibited in CD4^+^CD7^+^ T-lymphocytes and CD11b^+^ monocytes. At all PDT doses, there was significantly greater APO 2.7 positive cells among CD4^+^CD7^−^ malignant T-cells compared to either the nonmalignant or monocytic cell populations, whose levels of apoptosis ranged from 17.3% to 30.2% and from 26.4% to 37.2%, respectively. In the monocytic cell (CD11b^+^) population, the level of apoptosis increased slightly with each increment in Pc 4-PDT dose, but remained statistically insignificant in comparison to the levels in untreated monocytes. In addition, the degree of apoptotic monocytic cell death was not significantly different amongst the Pc 4 doses that were examined. Although a statistical difference was found between the monocytic cells treated with the 20 nM and 100 nM doses (*P* ≤ .003), the levels of apoptosis are far below those induced by Pc 4-PDT in the malignant T-cell population. A similar phenomenon was observed in the benign (CD4^+^CD7^+^) T-cell population, even though a statistical difference was observed between the cells treated with 70 nM and 100 nM (*P* ≤ .01). Levels of apoptosis induced in this population were again far below those in the malignant clones for each dose. This distinct feature seen in both benign cell populations suggests an apparent specificity of Pc 4 in the killing of targeted cancerous cells. Representative flow cytometric histograms for CD4^+^CD7^−^ T-cells, CD4^+^CD7^+^ T-cells, and CD11b^+^ monocytes from one patient with a diagnosis of SS are shown in Figures 2, 3, and 4, respectively.

**Figure 5:**
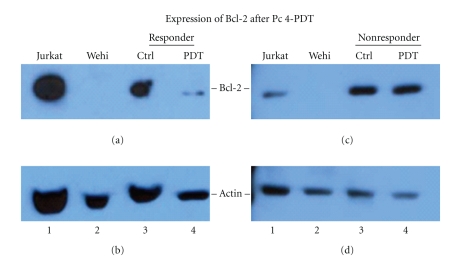
Representative Western blots of Bcl-2 expression after *in vivo* treatment with Pc 4-PDT. Skin biopsies of MF lesions were obtained from patients diagnosed with MF. This Western blot compares Bcl-2 expression in a single representative clinical responder to that of a single representative clinical nonresponder. The top panels in both (a) and (c) show the 26 KDal Bcl-2. (b) and (d) show bands corresponding to actin as loading control. For both the responder (a and b) and nonresponder blots (c and d), Lane 1 consists of Jurkat cells, a cell line known to have endogenous expression of Bcl-2. Lane 2 consists of Wehi cells, a mouse cell line known to have minimal endogenous Bcl-2 expression. Lanes 3 and 4 contain the extraction from the untreated and treated lesions, respectively. (a) The clinical responder patient shows significantly decreased Bcl-2 expression in treated lesions in comparison to Bcl-2 expression in untreated lesion. In Lane 2, there is no corresponding band, as expected. (b) In the clinical nonresponder patient, again, there is Bcl-2 expression detected in Jurkat cells and no expression in Wehi cells, as expected. However, in contrast to the clinical responder patient, the level of Bcl-2 expression in treated and untreated lesions of the nonresponder is similar. These results are supported by the level of actin protein, which shows approximately similar amounts of protein loaded.

As another indicator of PDT-induced photodamage leading to apoptosis, skin biopsies from MF patients were evaluated for the level of native Bcl-2 by western blot analysis (Figure 5). There was a significant decrease in Bcl-2 levels in response to Pc 4-PDT, but only in biopsies of those patients who demonstrated a partial response clinically. A representative blot from one such patient is shown in Figure 5(a). The level of Bcl-2 in the MF lesion of this patient after Pc 4-PDT treatment is notably reduced in comparison to the Bcl-2 level in the untreated lesion of the same patient. In contrast, the levels of Bcl-2 expression in both treated and untreated lesions are similar in the skin biopsy of the nonresponder (Figure 5(c)).

## 4. Discussion

The flow cytometry analysis and APO 2.7 staining of Pc 4-PDT-treated PBMCs from patients with SS demonstrated that Pc 4-PDT more effectively induces apoptosis in Sezary cells compared to other cell types. This mechanism of preferential killing of malignant lymphocytes remains enigmatic. One hypothesis is that the increased susceptibility of malignant cells may be related to changes involving the antiapoptotic protein Bcl-2, which has been found to be overexpressed in MF and SS [23], although subsets of patients lacking Bcl-2 have been reported [24]. This study also found that the response to Pc 4-PDT occurs in a Pc 4 dose-dependent manner. However, the increased level of apoptosis induced in CD4^+^CD7^−^ T-cells by the 100 nM dose compared to the 70 nM dose of Pc 4 was not statistically significant. It is possible that beyond the 70 nM dose the cells are already saturated with Pc 4 and so further increases do not result in increased apoptosis. Further experiments examining the effect of the intermediate doses of Pc 4 between 20 nM and 70 nM, as well as between 70 nM and 100 nM to optimize dosing are ongoing.

The Western blot analyses of MF skin lesions treated with Pc 4-PDT confirmed that this treatment is capable of reducing intracellular levels of Bcl-2 in malignant T-lymphocytes *in vivo*, consistent with our previous *in vitro* data [12]. The decrease in antiapoptotic Bcl-2 suggests that Pc 4-PDT enhanced apoptosis in these cells, which translated to clinical improvement. Conversely, when there was no change in Bcl-2 levels detected after Pc 4-PDT, no response was observed clinically. These observations support previous findings that photodamage to Bcl-2 is a critical component in the mechanism of Pc 4-PDT-mediated cell killing. More studies are being conducted using skin biopsies from T-cell driven pathologies such as CTCL to further assess the selectivity of cell killing after Pc 4-PDT. 

In summary, this study has shown that photodynamic therapy with Pc 4 can be delivered at a dose that induces killing of malignant lymphocytes in the peripheral blood and in tissue of patients with CTCL. Selective killing of malignant CD4^+^CD7^−^ T-cells over normal bystander cells was observed *in vitro*, which suggests that Pc 4-PDT can potentially be utilized as a target-specific anticancer therapy for malignancies such as CTCL. Therapies aimed at arresting neoplastic growth ideally should be able to eliminate aberrant cells while preserving the normal components of the tissue or organ. In theory, such a target-specific modality could result in clinical cure with limited adverse side effects, such as immunosuppression, severe tissue inflammation, and necrosis, which are commonly observed with other anti-cancer treatments like chemotherapy and radiation.
